# Binocular summation of visual acuity and contrast sensitivity in children with intermittent exotropia

**DOI:** 10.1186/s12886-023-02961-x

**Published:** 2023-06-01

**Authors:** Jun Liao, Yueping Li, Wei Zhang

**Affiliations:** 1 Chengdu AIDI Eye Hospital, Chengdu, China; 2 Pediatric Ophthalmology and Strabismus Department, Tianjin Eye Hospital, Affiliated Eye Hospital of Nankai University, Clinical College of Ophthalmology of Tianjin Medical University, Tianjin Key Laboratory of Ophthalmology and Vision Science, Tianjin, 300020 China

**Keywords:** Contrast sensitivity, Binocular summation, Visual acuity, Intermittent exotropia, Binocular vision

## Abstract

**Purpose:**

To investigate the binocular summation (BiS) of visual acuity (VA) and contrast sensitivity (CS) in children with intermittent exotropia (IXT) before and after surgery and to probe the relationship between the two BiS phenomena and corresponding influencing factors.

**Methods:**

This prospective study included 21 IXT children (11 males and 10 females; aged 6–13 years) who underwent strabismus surgery in Tianjin Eye Hospital from January to April 2022. The visual function was assessed preoperatively and 2.95 ± 0.14 months postoperatively, including monocular/ binocular visual acuity (MVA/BVA) at 100% contrast and 2.5% contrast as well as monocular/binocular contrast sensitivity (MCS/BCS), deviation, near and distant stereopsis, and fusion.

**Results:**

All patients had postoperative deviation ranging from 0 to -4 PD. Either preoperative or postoperative BVA at 2.5% contrast was superior to the MVA. The postoperative BiS at 2.5% contrast was significantly superior to the preoperative BiS for 2.5% contrast and postoperative BiS for 100% contrast (*P* < 0.05). Except for 3 c/d, the MCS and BCS at 6 c/d, 12 c/d and 18 c/d spatial frequencies were all notably improved postoperatively. The postoperative binocular summation ratio of CS (BSR) was highest while interocular difference ratio of CS (IOR) was the lowest at 6 c/d among 4 spatial frequencies. The deviation, distant and near stereopsis, and fusion performance were all remarkably improved after surgery (*p* = 0.001; *p* = 0.041; *p* = 0.000), all of which were not related to BVA at 2.5% contrast, BiS, BSC and BSR. The BCS at middle and high frequencies (6 c/ds, 12 c/ds, and 18 c/ds) was significantly negatively correlated with the BVA at 2.5% contrast, and BSR was irrelevant to the corresponding IOR across different spatial frequencies.

**Conclusion:**

BVA at low contrast and BCS examinations were not equivalent to stereopsis and fusion status, which contributed to the evaluation of binocular function in the real environment and in the different aspects. BVA in 2.5% contrast is related with BCS in moderate and high spacial frequencies (especially 18c/d) but BCS in 6c/d presents more binocular summation of contrast sensitivity. MCS, BCS and the BSR persist inhibition at 3c/d after surgery. The improvement of BCS is better than that of BSR to evaluate the binouclar function in IXT. Those two methods showed different sensitivities to impairment and rehabilitation of binocular summation and inhibition.

**Supplementary Information:**

The online version contains supplementary material available at 10.1186/s12886-023-02961-x.

## Introduction

Intermittent exotropia (IXT) is a common type of strabismus in children, which manifests the different degrees of binocular vision function loss at distance and at near [1]. Some patients gradually lost binocular vision and developed constant exotropia. Examinations such as worth-4 dot (W-4-D) test, striated glass test, and stereopsis tests at distance and near are commonly applied to evaluate the changes in binocular vision, but these examinations cannot fully reflect the changes of visual function in the actual daily environment. Binocular summation (BiS) refers to the superiority of binocular over monocular viewing on visual tasks, such as visual acuity (VA) and contrast sensitivity (CS), which involves the interaction in layer VI of the visual cortex and is easily presented at the low contrast [2]. The threshold of monocular contrast sensitivity (MCS) at low contrast is 1.5 times that of binocular contrast sensitivity (BCS) in healthy individuals [3]. BiS can be influnced by multiple factors such as age, interocular difference of visual acuity, and eye position [4–8]. Successful strabismus surgery can improve BiS in strabismus patients [9], which might be related to eye position control and stereopsis [10–13]. BCS in strabismus patients was significantly lower than monocular contrast sensitivity, especially at 6 cpd spatial frequency, which was not correlated with the degree of strabismus, gender, age, refraction error, stereopsis, and duration of IXT [14]. The BiS phenomenon and relevant mechanisms in IXT patients are still undetermined. The correlationship between BiS of VA and BiS of CS is not well probed. This study sought to characterize the changes in BiS for spatial resolution and contrast sensitivity in IXT patients before and after surgery and to identify the relationship between the two phenomena and corresponding influencing factors in IXT children.

## Subjects and methods

### Subjects

This study conformed to the Declaration of Helsinki, and all subjects signed informed consent. Twenty-one children with IXT who underwent strabismus surgery at Tianjin Eye Hospital from January to April 2022 were enrolled. The enrolled subjects, aged 6 to 13 years old [mean, 9.1 (2.1)], consisted of 11 males and 10 females. The average spherical equivalents were -1.83 (1.87)D( -6.0 to + 0.87 D) on the right eye and -1.34 (1.82) D ( -6.5 to + 0.75 D) on the left eye. We performed bilateral LR recession and R&R procedure, depends on the subtypes of IXT and deviation in the primary gaze. The postoperative assessments were taken at 2 to 3 months [mean 2.95 (0.74) months] after surgery.

Exclusion criteria: amblyopia, anisometropia (spherical anisometropia > 1.5D, cylindrical anisometropia > 1.0D), nystagmus, history of other ophthalmic surgeries or trauma, neurodevelopmental abnormalities, combined vertical strabismus (> 5PD), dissociated vertical deviation (DVD), and patients who did not cooperate with the examinations.

### Routine ocular examinations and binocular visual function examinations

All patients underwent the general ocular examinations by the same ophthalmologist, including slit-lamp and fundus examination, measurements of deviation with prisms and alternate occlusion in the primary position and nine-gaze before and after surgery, hole-in-the-card test for the dominant eye, W-4-D test, Titmus test for near stereopsis, and distant random dots stereograph (dRDS; P/N 1006, Vision assessment Corporation, Illinois, USA). Distant RDS results consisting of 63", 100", 200", 400", and unidentifiable (nil) were recorded as grades 1 to 5 in sequence. The central and peripheral fusion were evaluated using the W-4-D test at the distance of 3 m and 33 cm, respectively. The assessments of visual acuity, binocular vision and deviation were taken while patients was wearing spectacles with full refraction correction.

### High-contrast (100%) and low-contrast (2.5%) binocular visual acuity (BVA) examination and BiS assessment

LEA symbol VA test (Part B courtesy Good-Lite Company, Streamwood, and IL.) was carried out to examine monocular visual acuity (MVA) and BVA at 3 m in a dim room. The scores of visual acuity (log Mar) was recorded when patients was recognizing the full line.

The BiS score was calculated by the difference in the symbol lines between BVA and MVA of the better eye, which was classified as follows:(1) BiS > 1, BVA better than VA of the better eye > 1 line, called binocular summation; (2)-1 ≤ BiS ≤ 1, called equality; (3) BiS < -1, referring BVA worse than VA of the better eye > 1 line, named binocular inhibition.

### Examinations of MCS and BCS at different spatial frequencies and assessment of Binocular Summation Ratio (BSR) and Interocular difference (IOR) of CS

The patients were tested with full refractive correction at 2.5 m in a dim room before and after surgery, using the CSV-1000E device (VECTOR VISION, USA),with constant background light at 85 cd/cm^2^ without glare. The contrast sensitivity function (CSF) of BCS and MCS at different spatial frequencies (3, 6, 12 and 18 c/d) were measured, and the log value of the last correctly identified grating corresponding to each spatial frequency was recorded.

$$\mathrm{Binocular\;summation\;ratio}\left(\mathrm{BSR}\right)=\frac{\mathrm{BCS}}{\mathrm{MCS\;of\;the\;better\;eye}}$$

BSR was classified into: (1) BSR > 1, named binocular summation; 2) BSR = 1, named equality; 3) BSR < 1, named binocular inhibition.

$$\mathrm{Interocular\;difference}\left(\mathrm{IOR}\right)\mathrm{of\;CS}=\frac{\mathrm{CS\;of\;the\;non}-\mathrm{dominant\;eye}}{\mathrm{CS\;of\;the\;better\;eye}}$$

### Statistical analysis

All data were analyzed using SPSS, version 20.0 (IBM, Armonk, NY, USA). Comparisons of preoperative and postoperative data were conducted by paired *t* test or Wilcoxon rank test. Data between two groups were compared using the analysis of variance test and chi-square test or Fisher's exact test. Pearson’s correlation method was used to analyze the correlations among different examination parameters. The confidence interval (CI) used in this study was 95% with an alpha of 0.05 (a = 0.05).

## Results

### Comparisons of preoperative and postoperative deviation, stereopsis, and fusion statuses in IXT patients

The postoperative deviation of IXT patients was significantly reduced as compared to the preoperative deviation (-0.82 ± 1.852 [-4 ~ 0] PD *vs*. -34.86 ± 13.99 [-20 ~ -72] PD; *t* = -10.573, *p* = 0.000).

**Fig. 1:**
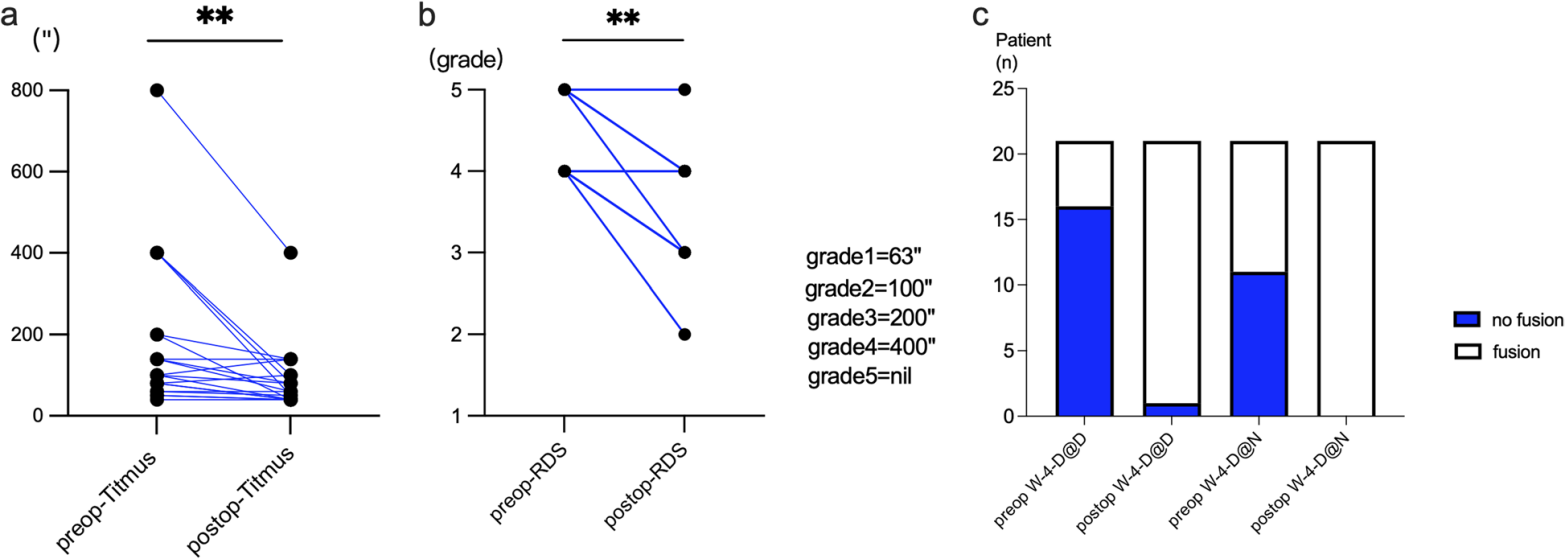
Preoperative and postoperative stereopsis and fusion in IXT children. **a** Titmus test. **b** Distant RDS,grade1,63″; 2,100″; 3,200″; 4,400″ and 5, nil. **c** Fusion tested by W-4D at near (N) and at distance (D). *, *P* < 0.05, **, *P* < 0.01

The results of the Titmus test after surgery (40″ to 800″; mean 90.48″ ± 80.34″) revealed a significant improvement of near stereopsis than that before surgery (40″ to 400″; mean 181″ ± 187.31″)(*t* = 3.062, *P* = 0.0032) (Fig. 1a). 16 patients (76.19%) achieved the improvements of Titmus tests at different extents following surgery.

According to the RDS scale of distant stereopsis (Fig. 1b), the median postoperative RDS score was 4, (3 to 5), which was noticeably increased versus the mean preoperative RDS score 4,(4 to5) (Wilcoxon, *P* = 0.02). The distant stereopsis was improved at different extents in 12 individuals (57.14%).The results of the W-4-D examination exhibited that 5 patients (23.8%) had a central fusion before surgery, the number of which was increased to 20 (95.23%) after surgery (F = 22.862, *P* = 0.000). Additionally, the number of patients with peripheral fusion was increased from 10 (47.62%) to 21 (100%) after surgery (F = 14.807, *P* = 0.000). Both the two fusion statuses were significantly improved (Fig. 1c).

### Preoperative and postoperative BVA, MVA, and BiS at 100% and 2.5% contrast in IXT patients

**Fig. 2:**
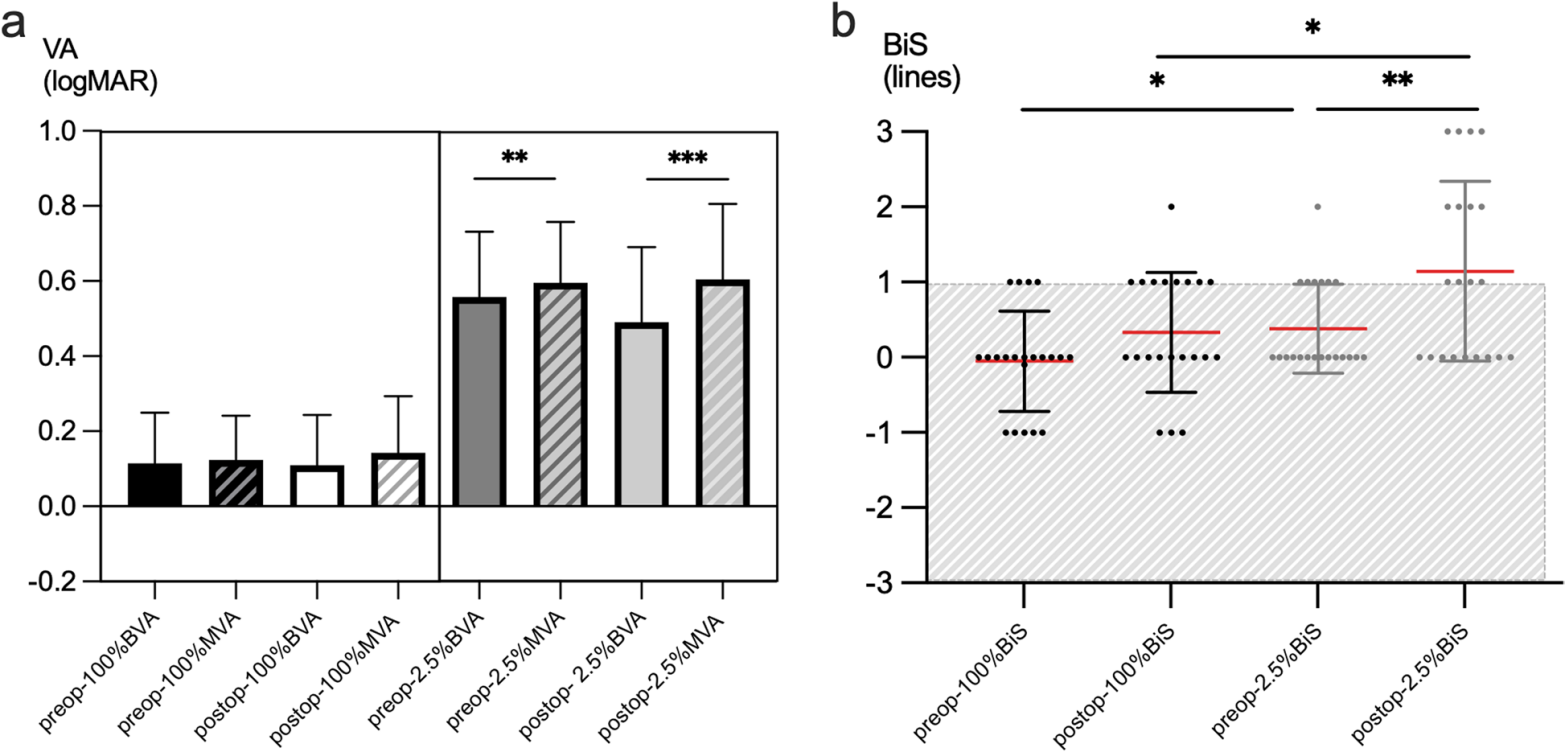
Preoperative and postoperative binocular/monocular visual acuity and binocular summation at 100% contrast and at 2.5% contrast. **a** Binocular/monocular visual acuity. **b** Binocular summation. *, *P* < 0.05, **, *P* < 0.01, ***, *P* < 0.001

The preoperative and postoperative BVA, MVA, and BiS values at 100% contrast and 2.5% contrast were shown in Table 1 and Fig. 2.

**Table 1:** The preoperative and postoperative BVA, MVA of better eye, and BiS values at 100% contrast and 2.5% contrast

	At 2.5% contrast (logMAR)			At 100% contrast (logMAR)		
	BVA	MBA	BIS	BVA	MBA	BIS
pre-op	0.56 ± 0.17	0.6 ± 0.163	0.038 ± 0.59	0.114 ± 0.14	0.124 ± 0.12	0.1 ± 0.06
Post-op	0.49 ± 0.2	0.6 ± 0.6	0.114 ± 0.12	0.11 ± 0.13	0.14 ± 0.15	0.33 ± 0.80
t	1.214	0.175	2.685	0.181	0.698	0.925
p	0.239	0.863	0.014*	0.858	0.493	0.116

*BVA* Binocular visual acuity, *MVA* unilateral visual acuity of the better eye, *BIS* Binocualr summation

^*^p < 0.05

BVA at 2.5% contrast was significantly superior to MVA both before surgery (*t* = -2.961, P = 0.008) and after surgery (*t* = -4.382, *P* = 0.000) (Fig. 2a). No significant difference was detected between preoperative BiS at 2.5% contrast and that at 100% contrast (*t* = 1.430, *P* = 0.160), while the postoperative BiS at 2.5% contrast was superior to that at 100% contrast (*t* = 2.583, *P* = 0.014). Additionally, the BiS at 2.5% contrast achieved a significant improvement postoperatively (*t* = 2.685, *P* = 0.014) (Fig. 2b).

### Preoperative and postoperative MCS, BCS, and BSR at varied spatial frequencies in IXT patients

**Fig. 3:**
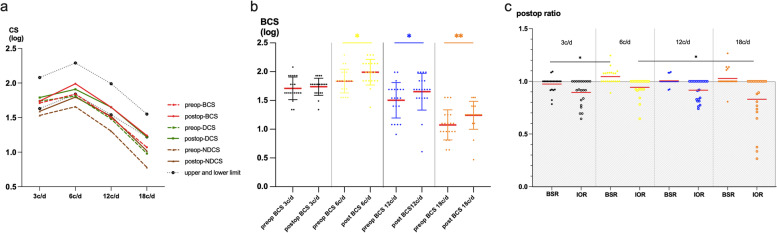
Preoperative and postoperative contrast sensitivity (CS). **a** Binocular CS, dominant eye CS and nondominant eye CS before and after surgery. **b** Comparisions of BCS between preoperative value and postoperative value. **c** Postoperative BSR and IOR of CS at different spacial frequencies. *, *P* < 0.05, **, *P* < 0.01, ***, *P* < 0.001

Table 2 and Fig. 3a,b showed the BCS and MCS of the dominant eye and the non-dominant eye before and after surgery. Except for 3 c/d, the BCS values and MCS of the dominant eye and the non-dominant eye values at 6, 12, and 18 c/d were all significantly improved following surgery.

**Table 2:** CS values of both eyes, the dominant eye, and the non-dominant eye before and after surgery

	3c/d (log)			6c/d (log)			12c/d (log)			18c/d (log)		
	BCS	DCS	NDCS	BCS	DCS	NDCS	BCS	DCS	NDCS	BCS	DCS	NDCS
Pre-op	1.71 ± 0.20 (1.34 ~ 2.08)	1.74 ± 0.23 (1.17 ~ 2.08)	1.53 ± 0.27 (1.00 ~ 1.93)	1.84 ± 0.20 (1.55 ~ 2,29)	1.81 ± 0.22 (1.38 ~ 2.29)	1.66 ± 0.29 (1.21 ~ 2.29)	1.50 ± 0.31 (0.91 ~ 1.99)	1.48 ± 0.29 (0.91 ~ 1.99)	1.30 ± 0.33 (0.91 ~ 1.99)	1.10 ± 0.26 (0.64 ~ 1.55)	0.96 ± 0.32 (0.47 ~ 1.55)	0.78 ± 0.28 (0.47 ~ 1.25)
Post-op	1.74 ± 0.15 (1.34 ~ 1.93)	1.79 ± 0.14 (1.49 ~ 2.08)	1.60 ± 0.19 (1.34 ~ 1.93)	1.99 ± 0.22 (1.38 ~ 2.29)	1.91 ± 0.18 (1.55 ~ 2.29)	1.80 ± 0.25 (1.21 ~ 2.29)	1.65 ± 0.32 (0.61 ~ 1.99)	1.65 ± 0.31 (0.61 ~ 1.99)	1.52 ± 0.30 (0.91 ~ 1.99)	1.24 ± 0.24 (0.47 ~ 1.55)	1.22 ± 0.26 (0.47 ~ 1.55)	1.02 ± 0.36 (0.17 ~ 1.55)
t	0.6736	0.9736	-0.892	2.702	2.431	-2.316	2.711	2.674	-3.746	3.129	4.357	-3.151
p	0.508	0.342	0.383	0.014*	0.0246*	0.031*	0.014*	0.0146*	0.001*	0.0053*	0.0003*	0.005*

*BCS* Binocular contrast sensitivity, *DCS* Contrast sensitivity of dominant eye, *NDCS* Contrast sensitivity of non-dominant eye

^*^p < 0.05

After surgery, BSR at 3 c/d was less than 1, while BSR values at other spatial frequencies were greater than 1, demonstrating a binocular summation phenomenon. There were a significant difference regarding BSR among different spatial frequencies (ANOVA, F = 3.854, *P* = 0.012). Further, Tukey's multiple comparisons test showed significant differences between those values at 6 c/d and 3 c/d (*P* = 0.005). The postoperative IOR values were statistically different among different spatial frequencies (ANOVA, F = 3.790, *P* = 0.030) and further Tukey's multiple comparisons test demonstrated noticeable differences between those at 6 c/d and 18 c/d (*P* = 0.017) (Table 3, Fig. 3c). At 6 c/d frequency, the postoperative BSR was the highest while the IOR was the lowest.

**Table 3:** Postoperative BSRs and IORs at different spatial frequencies

Post-op	3c/d (log)	6c/d (log)	12c/d (log)	18c/d (log)	F	P
BSR	0.97 ± 0.07	1.04 ± 0.07	1.00 ± 0.04	1.03 ± 0.88	3.854	0.012*
IOR	0.90 ± 0.12	0.94 ± 0.91	0.92 ± 0.99	0.83 ± 0.24	3.790	0.030*

*BSR* Binocular summation ratio of CS, *IOR* Interocular difference of CS

^*^p < 0.05

### Correlation analyses of BiS of visual acuity and contrast sensitivity

**Fig. 4:**
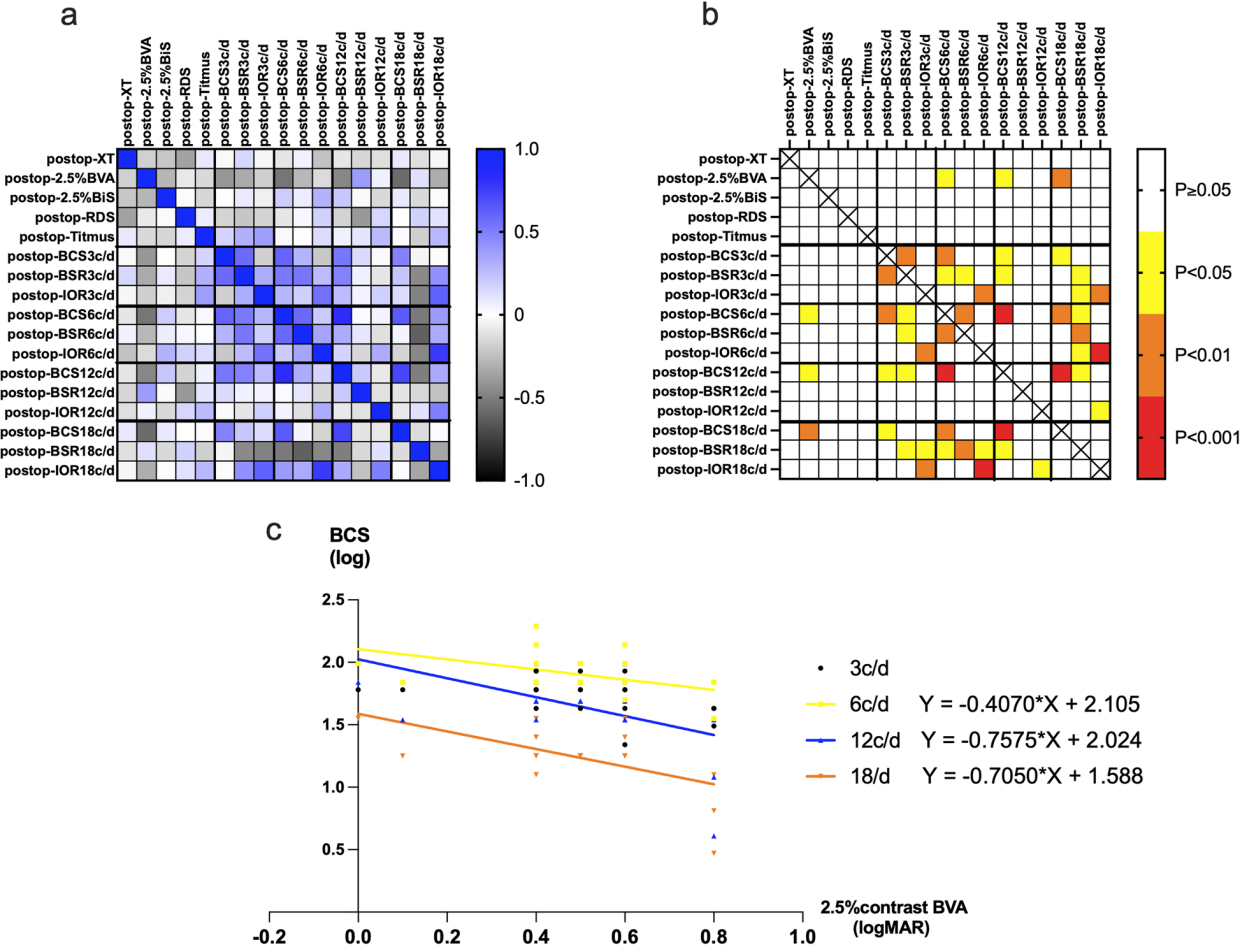
Correlation analysis among the different measurments. **a** The heat map of pearson correlation coefficents. **b** The heat map of *P* values. **c** The correlations between BVA at 2.5% contrast and BCS at 4 spacial frequencies and regression equations

There was no significant correlations of postoperative deviation or distant/near stereopsis with BiS of visual acuity and contrast sensitivity (Fig. 4a,b). Postoperative BVA at 2.5% contrast was significantly negatively correlated with postoperative BCS at 6 c/d, 12 c/d, and 18 c/d (*r* = -0.538, *P* = 0.012; *r* = -0.473, *P* = 0.03; *r* = -0.579, *P* = 0.006), except for 3c/d. (Fig. 4a,b); in other words, the better BVA at 2.5% contrast, the higher the BCS value. Their fitting equation is depicted in Fig. 4c. The correlation analyses of postoperative BCS and BSR at varied spatial frequencies only showed positive correlations between BCS and BSR at 3 c/d and 6 c/d. There was no correlation of IOR with BCS and BSR at each spatial frequency after surgery.

Also, BiS for 2.5% contrast and 100% contrast in patients with central fusion was statistically similar to those in patients without central fusion (*P* > 0.05). BSR values at 4 spatial frequencies also exhibited no significant differences between patients with and without central fusion (*P* > *0.05*). Additionally, BiS for 2.5% contrast and 100% contrast showed no noticeable differences between patients with and without peripheral fusion (*P* > 0.05), and BSR values at spatial frequencies were statistically similar between patients with and without peripheral fusion (*P* > *0.05*).

The raw measurements for the 21 individuals were shown in the supplementary Table 1.

## Discussion

Strabismus affects binocular vision as well as perception, fusion, and stereopsis. People perceive objects in daily life using both eyes, and the BiS value of a healthy individual is close to 1.4 [15]. The decline in vision-specific quality of life is associated with decreased vision under low-contrast conditions [16, 17]. Binocular neurons were reduced in patients with strabismus, and the magnitude of the BiS was decreased and close to the VA of better eye in the presence of a large interocular VA difference [3]. A prior study demonstrated that patients with strabismus had a reduction in BiS at 2.5% contrast and 1.25% contrast or even binocular inhibition, indicating a more serious impact of strabismus on binocular vision than previously recognized [18]. Decreased CS in IXT patients may be related to poor control and stereopsis [11, 12], and may also be independent of angle of deviation, gender, age, stereoscopic acuity and duration of IXT [14]. And the significant reduction in BiS at low contrast was regarded to correlate with the poor control of IXT patients, rather than near and distant stereopsis [19].

Our previous study suggested that BVA at low contrast was significantly improved after strabismus surgery and the number of IXT patients with the BiS was increased and that of binocular inhibition was decreased, which was related to obtaining postoperative central fusion and better recovery of distance stereopsis in some extent [9]. The present study revealed successfully corrected eye position and improved postoperative high- and low-contrast BiS in IXT patients, particularly at 2.5% contrast. However, BiS and BVA values exhibited no linear correlation with fusion and stereopsis. The higher values reported in this study than previous report, that might be related to the enrollment of only IXT patients and the age of the patients. Additionally, our study found that preoperative BVA at 2.5% contrast was better than the VA of the dominant eye, and preoperative BiS at 2.5% contrast was higher than that at 100%. The postoperative BiS at 2.5% contrast was significantly improved as compared to the preoperative BiS at 2.5% contrast and postoperative BiS at 100% contrast. We considered that the visual pathways for low contrast may be influenced comparatively later in IXT.

In the previous studies, the BSR was usually adopted as the evaluation parameter for the BiS phenomenon of CS, but the calculation methods were different such as BSR = BCV/MCV [8, 10, 20], or BSR = BCV^2^ /(RCV^2^ + LCV^2^) [12]. There also existed differences in evaluation methods such as sinusoidal grating [12, 20], Mars contrast sensitivity test [8, 10], etc. The animal experiments demonstrated that neurons in the lateral geniculate nucleus of strabismic amblyopia were only damaged at high spatial frequencies [21]. An existing study revealed that the BSR of IXT patients was significantly lower than that of the healthy controls at low spatial frequencies (1.5 c/d and 3 c/d), but no significant difference was noted at other spatial frequencies 6 c/d, 12 c/d, and 18 c/d [12]. CS was improved in bright conditions following surgical treatment of IXT patients while CS was decreased significantly in response to postoperative overcorrection [22]. CSF of amblyopia patients with fusion function was impaired at intermediate and high frequencies, presenting a higher proportion of BiS than strabismus patients without fusion function [8]. BSR of IXT patients was temporarily decreased 1 month postoperatively, which might be due to postoperative foreign body sensation, lacrimation, and conjunctival edema, and then it returned to the preoperative level 3 months after surgery [10].

In our series, the preoperative CS value in IXT children was decreased mainly at medium and high spatial frequencies (6c/d, 12c/d, 18c/d), which was similar to the finding reported in the investigation of strabismus amblyopia [23–25]. Those values were restored to normal levels postoperatively but CS of nondominant eye was still lower than the mean CS of 10-year-old healthy children reported previously [26]. BSR at 3 c/d persisted binocular inhibition at the low frequency even after surgery. The most significant improvement was achieved postoperatively in the BSR and BCS at 6 c/d in IXT children. Larsson found that the VA value was correlated with CS at medium and high spatial frequencies (6 c/d, 12 c/d, and 18 c/d) rather than CS at 3 c/d in 217 healthy children [26], which is consistent with our findings. In addition, postoperative BCS in our study shared no correlation with stereopsis and fusion function, which was similar to Chung’s report [14].

Human visual system exists the multiple pathways, each of which is only related to its specific narrow-band spatial frequency and directionality [27]. Cells in area 17 in the visual cortex are inclined to respond to higher spatial frequencies, while those in area 18 are likely to respond to lower spatial frequencies [27]. Low spatial frequency involved the macrocellular pathway [12]. Multiple visual mechanisms might be implicated in the tests for threshold level and super-threshold level [20]. We suggest that the low-contrast BVA method was superior in presenting the binocular summation, and CS tests showed the different sensitivity to binocular summation across different spatial frequencies. Both supplemented the clinical assessment methods for binocular visual performance.

However, the study had certain limitations, such as a small sample size, short duration of follow-up, and absence of a control group. Additionally, as all patients gained peripheral fusion after surgery, and there was only one patient without central fusion, so we failed to statistically analyze the correlations of postoperative BiS and BSR with fusion function. Thus, a large sample scale and long-term follow-up are needed to further study in IXT children.

In conclusions, binocular VA and CS reflect the details of BiS at different visual pathways and different aspects, which are not related to stereopsis and fusion function and could not be substituted. Although BiS at 2.5% contrast is associated with BCS at 6/12 /18 c/d, they cannot replace each other. In the future, more attention should be paid to multi-dimensional binocular visual performance.

## Supplementary Information

**Additional file 1: Supplementary Table 1.** The raw measurements for the 21 individuals.

## Data Availability

All data generated or analysed during this study are included in this published article and its supplementary information files.
