# Supplementary material for: Binocular summation of visual acuity and contrast sensitivity in children with intermittent exotropia
Source: BMC Ophthalmol. 2023 Jun 1;23:245. doi: 10.1186/s12886-023-02961-x (PMC10233972; doi:10.1186/s12886-023-02961-x)
Supplement: Supplementary file 1 — Additional file 1: Supplementary Table 1. The raw measurements for the 21 individuals. [file 12886_2023_2961_MOESM1_ESM.docx]

supplementary Table 1. The raw measurements for the 21 individuals

| No. | preop-B3c/d | preop-U3c/d | preop-BSR3c/d | postop-B3c/d | postop-U3c/d | postop-BSR3c/d | preop-B6c/d | preop-U6c/d | preop-BSR6c/d | postop-B6c/d | postop-U6c/d | postop-BSR6c/d |
| --- | --- | --- | --- | --- | --- | --- | --- | --- | --- | --- | --- | --- |
| 1 | 1.49 | 1.63 | 0.914110429 | 1.34 | 1.63 | 0.82208589 | 1.84 | 1.7 | 1.082352941 | 1.84 | 1.84 | 1 |
| 2 | 1.63 | 1.63 | 1 | 1.78 | 1.78 | 1 | 1.99 | 1.84 | 1.081521739 | 2.14 | 1.99 | 1.075376884 |
| 3 | 2.08 | 2.08 | 1 | 1.93 | 1.93 | 1 | 2.14 | 1.99 | 1.075376884 | 2.14 | 2.14 | 1 |
| 4 | 1.78 | 1.93 | 0.922279793 | 1.63 | 2.08 | 0.783653846 | 1.84 | 1.7 | 1.082352941 | 1.38 | 1.55 | 0.890322581 |
| 5 | 1.63 | 1.63 | 1 | 1.63 | 1.63 | 1 | 1.84 | 1.84 | 1 | 1.84 | 1.84 | 1 |
| 6 | 1.93 | 2.08 | 0.927884615 | 1.78 | 1.78 | 1 | 1.99 | 1.99 | 1 | 2.14 | 1.99 | 1.075376884 |
| 7 | 1.93 | 1.93 | 1 | 1.93 | 1.93 | 1 | 1.7 | 1.84 | 0.923913043 | 2.29 | 1.99 | 1.150753769 |
| 8 | 1.78 | 1.78 | 1 | 1.93 | 1.93 | 1 | 1.84 | 1.84 | 1 | 2.14 | 2.14 | 1 |
| 9 | 1.63 | 1.78 | 0.915730337 | 1.78 | 1.78 | 1 | 1.55 | 1.38 | 1.123188406 | 1.99 | 1.99 | 1 |
| 10 | 1.78 | 1.93 | 0.922279793 | 1.78 | 1.93 | 0.922279793 | 2.14 | 1.99 | 1.075376884 | 2.14 | 1.99 | 1.075376884 |
| 11 | 1.93 | 1.93 | 1 | 1.78 | 1.78 | 1 | 1.84 | 2.29 | 0.80349345 | 2.29 | 2.29 | 1 |
| 12 | 1.63 | 1.78 | 0.915730337 | 1.49 | 1.49 | 1 | 1.55 | 1.55 | 1 | 1.7 | 1.55 | 1.096774194 |
| 13 | 1.63 | 1.49 | 1.093959732 | 1.78 | 1.63 | 1.09202454 | 1.55 | 1.55 | 1 | 1.84 | 1.84 | 1 |
| 14 | 1.34 | 1.34 | 1 | 1.63 | 1.63 | 1 | 1.55 | 1.55 | 1 | 1.99 | 1.84 | 1.081521739 |
| 15 | 1.34 | 1.17 | 1.145299145 | 1.78 | 1.78 | 1 | 1.7 | 1.55 | 1.096774194 | 1.84 | 1.7 | 1.082352941 |
| 16 | 1.93 | 1.78 | 1.084269663 | 1.78 | 1.93 | 0.922279793 | 1.7 | 1.7 | 1 | 1.84 | 1.84 | 1 |
| 17 | 1.63 | 1.63 | 1 | 1.78 | 1.78 | 1 | 1.84 | 1.84 | 1 | 1.99 | 1.84 | 1.081521739 |
| 18 | 1.93 | 1.93 | 1 | 1.63 | 1.78 | 0.915730337 | 2.29 | 2.14 | 1.070093458 | 1.84 | 1.84 | 1 |
| 19 | 1.63 | 1.63 | 1 | 1.63 | 1.78 | 0.915730337 | 1.84 | 1.84 | 1 | 2.14 | 1.99 | 1.075376884 |
| 20 | 1.63 | 1.78 | 0.915730337 | 1.78 | 1.78 | 1 | 1.84 | 1.99 | 0.924623116 | 1.99 | 1.99 | 1 |
| 21 | 1.63 | 1.78 | 0.915730337 | 1.93 | 1.78 | 1.084269663 | 1.99 | 1.84 | 1.081521739 | 2.29 | 1.84 | 1.244565217 |

| No. | preop-B12c/d | preop-U12c/d | preop-BSR12c/d | postop-B12c/d | postop-U12c/d | postop-BSR12c/d | preop-B18c/d | preop-U18c/d | preop-BSR18c/d | postop-B18c/d | postop-U18c/d | postop-BSR18c/d |
| --- | --- | --- | --- | --- | --- | --- | --- | --- | --- | --- | --- | --- |
| 1 | 1.4 | 1.25 | 1.12 | 1.54 | 1.54 | 1 | 1.1 | 0.96 | 1.145833333 | 1.25 | 1.25 | 1 |
| 2 | 1.69 | 1.54 | 1.097402597 | 1.69 | 1.84 | 0.918478261 | 0.96 | 1.1 | 0.872727273 | 1.55 | 1.55 | 1 |
| 3 | 1.69 | 1.54 | 1.097402597 | 1.84 | 1.84 | 1 | 1.25 | 1.25 | 1 | 1.4 | 1.25 | 1.12 |
| 4 | 1.08 | 1.25 | 0.864 | 0.61 | 0.61 | 1 | 0.47 | 0.17 | 2.764705882 | 0.81 | 0.64 | 1.265625 |
| 5 | 1.54 | 1.54 | 1 | 1.54 | 1.54 | 1 | 0.96 | 1.1 | 0.872727273 | 1.1 | 1.1 | 1 |
| 6 | 1.54 | 1.99 | 0.773869347 | 1.84 | 1.99 | 0.924623116 | 1.55 | 1.4 | 1.107142857 | 1.55 | 1.55 | 1 |
| 7 | 1.69 | 1.99 | 0.849246231 | 1.99 | 1.99 | 1 | 0.96 | 0.81 | 1.185185185 | 1.1 | 1.1 | 1 |
| 8 | 1.99 | 1.69 | 1.177514793 | 1.99 | 1.84 | 1.081521739 | 0.96 | 0.96 | 1 | 1.55 | 1.4 | 1.107142857 |
| 9 | 1.4 | 1.25 | 1.12 | 1.69 | 1.69 | 1 | 1.25 | 0.81 | 1.543209877 | 1.25 | 1.25 | 1 |
| 10 | 1.69 | 1.69 | 1 | 1.69 | 1.69 | 1 | 0.96 | 1.25 | 0.768 | 1.4 | 1.4 | 1 |
| 11 | 1.69 | 1.69 | 1 | 1.99 | 1.99 | 1 | 1.25 | 1.4 | 0.892857143 | 1.4 | 1.4 | 1 |
| 12 | 1.08 | 1.08 | 1 | 1.08 | 1.08 | 1 | 0.64 | 0.64 | 1 | 0.47 | 0.47 | 1 |
| 13 | 0.91 | 0.91 | 1 | 1.69 | 1.69 | 1 | 0.81 | 0.47 | 1.723404255 | 1.25 | 1.25 | 1 |
| 14 | 1.08 | 1.25 | 0.864 | 1.54 | 1.54 | 1 | 0.96 | 0.81 | 1.185185185 | 1.25 | 1.25 | 1 |
| 15 | 1.4 | 1.4 | 1 | 1.54 | 1.54 | 1 | 1.1 | 0.81 | 1.358024691 | 1.25 | 1.1 | 1.136363636 |
| 16 | 1.84 | 1.25 | 1.472 | 1.69 | 1.69 | 1 | 0.81 | 0.47 | 1.723404255 | 1.25 | 1.25 | 1 |
| 17 | 1.08 | 1.25 | 0.864 | 1.54 | 1.54 | 1 | 1.1 | 0.81 | 1.358024691 | 1.25 | 1.1 | 1.136363636 |
| 18 | 1.99 | 1.84 | 1.081521739 | 1.99 | 1.84 | 1.081521739 | 1.55 | 1.25 | 1.24 | 1.25 | 1.25 | 1 |
| 19 | 1.54 | 1.4 | 1.1 | 1.69 | 1.69 | 1 | 1.1 | 0.96 | 1.145833333 | 1.25 | 1.25 | 1 |
| 20 | 1.54 | 1.69 | 0.911242604 | 1.69 | 1.69 | 1 | 1.1 | 1.25 | 0.88 | 1.25 | 1.25 | 1 |
| 21 | 1.69 | 1.69 | 1 | 1.84 | 1.69 | 1.088757396 | 1.55 | 1.55 | 1 | 1.25 | 1.55 | 0.806451613 |

Preop，preoperative； postop， post operative； B, bilateral; U, unilateral
